# Mortality risk prediction in octogenarians undergoing emergency colorectal surgery: a tertiary center experience and systematic review of the literature

**DOI:** 10.3389/fsurg.2025.1649766

**Published:** 2025-07-31

**Authors:** Marco Brolese, Arianna Vittori, Matteo Todisco, Nadine Zuin, Vanessa Cusano, Valeria Valli, Lorenzo Vallese, Nicola Baldan, Michele Valmasoni, Gianfranco Da Dalt, Alberto Friziero

**Affiliations:** ^1^ 1st General Surgery, Acute Care Surgery Unit, Department of Surgery, Oncology and Gastroenterology, University of Padova, Azienda Ospedale Università Padova, Padova, Italy; ^2^ Department of Radiology, Azienda Ospedale Università Padova, University of Padova, Padova, Italy

**Keywords:** emergencies, acute care surgery, colectomy, octogenarians, risk factors, mortality

## Abstract

**Objective:**

Surgical colonic emergencies frequently occur in elderly patients. In these cases a comprehensive preoperative assessment is crucial to tailor surgical invasiveness to individual risk profiles and potentially improve clinical outcomes. The aim of our study was to identify predictors of in-hospital mortality in octogenarians undergoing emergency colorectal resections, and compare the short-term outcomes between elderly and younger patients.

**Methods:**

This study included patients who underwent emergency colorectal resections at Our Department between January 2020 and December 2024. Exclusion criteria were age <18 years and palliative surgery. Patients were stratified into two cohorts: octogenarians (≥80 years, Group 1) and patients aged <80 years (Group 2). Baseline characteristics, perioperative variables, and short-term outcomes were compared and analyzed. A systematic review (PROSPERO: CRD420251050770) was conducted to identify the studies evaluating outcomes of emergency colorectal resections in octogenarians. MEDLINE (via PubMed), EMBASE, and EBSCOhost were searched from database inception to April 2025.

**Results:**

Group 1 and Group 2 included 82 and 130 patients with median ages of 84 and 67 years, respectively. The in-hospital mortality rate was 24% in octogenarians and 8% in younger patients (*p* < 0.001). Multivariate logistic regression identified hyperlactatemia as an independent negative prognostic factor for in-hospital mortality in octogenarians (*p* = 0.01). Through a systematic review of the literature we identified 12 publications, and the mortality rate ranged between 7.0 and 37.8%.

**Conclusions:**

Early identification of prognostic factors can improve clinical outcome in emergency scenarios. Our systematic review, the first reported in the literature, provides a comprehensive perspective in this field.

## Introduction

Advances in biomedical sciences over the past century have led to a steady increase in life expectancy, particularly in developed countries (1). In Italy and other Western countries, the proportion of elderly individuals has increased as birth rates decline, resulting in a steady rise in average age (2). This demographic shift has been accompanied by a growing demand for both social and healthcare services, necessitating their systematic enhancement and expansion (3). In this context, octogenarians now represent a substantial proportion of the general population and frequently require hospital admission and intensive care support (4, 5). Elderly patients are typically more frail and often suffer from multiple comorbidities, which may limit the feasibility of appropriate medical or surgical treatments (6). Accordingly, such studies have identified age as a negative prognostic factor, contributing to increased rates of postoperative morbidity and mortality (7). To mitigate the adverse impact of age, a comprehensive clinical and preoperative assessment is crucial. In the surgical field this challenge applies to both elective and emergency procedures, however, emergency settings often allow less time for thorough preoperative evaluation and optimization (8, 9). Furthermore, emergency surgery is consistently associated with higher morbidity and mortality rates compared to elective procedures, ranging from 33% to 64% and 20% to 34%, respectively (10). Thus, geriatric surgery, especially in emergency scenarios, represents a highly relevant and pressing issue in modern medicine. Many colonic emergencies, including colorectal cancer, diverticulitis, and colonic ischemia, are age-related conditions with a high prevalence among octogenarians (11, 12). In these situations, emergency surgeons are often required to decide between a palliative ostomy and a more complex procedure such as a visceral resection, whenever feasible. Emergency colorectal resections in elderly patients are well known to carry a significant risk of postoperative complications, with in-hospital mortality rates ranging from 15.6% to 37.8% (13, 14). Therefore, this scenario warrants further investigation to contribute to better predicting clinical outcomes in this patient population, and consequently, to improve their clinical outcomes. The primary aim of our study is to identify predictors of short-term mortality in octogenarians undergoing emergency colorectal resections. Additionally, we conducted a systematic review to evaluate the outcome of emergency colorectal resection in octogenarians highlighting key factors essential for appropriate patient selection.

## Methods

### Study population

This single-centre retrospective study analyzed. data from a prospectively maintained database. Adult patients (>18 years old) who underwent colorectal resection at the Department of Acute Care Surgery, Azienda Ospedale–Università di Padova (AOUP), between January 2020 and December 2024 were included. Patients were excluded if they received palliative interventions such as colostomy without colonic resection. Patients were divided in two groups: patients 80 years or older (Group 1) and those younger than 80 years (Group 2). This study was conducted in accordance with the principles of the Declaration of Helsinki. Given the retrospective nature of the study, ethical committee approval was not required in accordance with institutional policies.

Demographic variables included sex and age. Baseline comorbidities consisted of cardiovascular, hepatic, pulmonary, renal, and cerebrovascular diseases, hematological and metabolic disorders, oncological history and the use of antithrombotic drugs.

### Preoperative workup

Comorbidity burden was assessed using the age-adjusted Charlson Comorbidity Index (CCI) (15), the performance status was evaluated using the Eastern Cooperative Oncology Group Performance Status (ECOG-PS) (16), and functional status was determined according to the American Society of Anesthesiologists (ASA) classification (17). The Portsmouth Physiological and Operative Severity Score for the enUmeration of Mortality and Morbidity (P-POSSUM) was calculated for each patient to estimate the risk of in-hospital mortality. The Systemic Inflammatory Response Syndrome (SIRS) criteria were also applied to assess the degree of physiological compromise (18). All preoperative laboratory values, including hemoglobin, white blood cell count, C-reactive protein, lactate, creatinine, and albumin, were recorded based on the last available measurements prior to surgery. Indications for admission were categorized as visceral perforation, colonic obstruction, ischemia, or bleeding.

### Surgical management

Timing of surgery was categorized based on time from admission as emergent (within 12 h), urgent (12–72 h), or delayed (more than 72 h). Specifically, delayed surgery, defined as operative intervention performed more than 72 h after admission, was observed in patients who initially presented with stable or permissive clinical conditions but subsequently experienced clinical deterioration during hospitalization. The surgical approach was classified as open, laparoscopic, or converted to open surgery, defined as an intraoperative switch from a minimally invasive to an open surgical technique. Surgical interventions included conventional colonic resections, with or without the creation of protective stomas, such as right or left colectomy, total colectomy, sigmoidectomy, Hartmann's procedure, ileocecal resection, and anterior rectal resection. Damage control procedures, such as staged laparotomy and placement of abdominal Vacuum-Assisted Closure (VAC), were also performed when indicated. Malignant lesions were staged according to the latest American Joint Committee on Cancer—Tumor, Node, Metastasis (AJCC-TNM) staging system (19)*.* Intraoperative blood transfusion and use of vasoactive agents were documented.

### Postoperative outcomes

Postoperative complications were assessed according to the Clavien–Dindo (CD) classification (20) and were defined as anastomotic leak, intra-abdominal collection, fistula formation, bleeding requiring transfusion, prolonged ileus (>7 days), and wound infections. Medical complications included cardiovascular, pulmonary, renal and cerebrovascular events (21). Reoperation was categorized into two groups: planned reoperation (second-look procedure in case of a staged laparotomy) and reoperation for complications. Length of stay (LOS) was reported as median and interquartile range (IQR). Thirty-day mortality was defined as all-cause death within 30 days of surgery, while in-hospital mortality referred to any death occurring during the index hospitalization, regardless of its duration.

### Outcomes and objectives

The primary outcome was in-hospital mortality in patients aged 80 years or older. The primary objective was to identify preoperative factors predictive of in-hospital mortality in this population. Secondary outcomes included comparisons between Group 1 and Group 2 in terms of in-hospital mortality, length of stay (LOS), and postoperative complications.

### Systematic literature review

**Figure 1:**
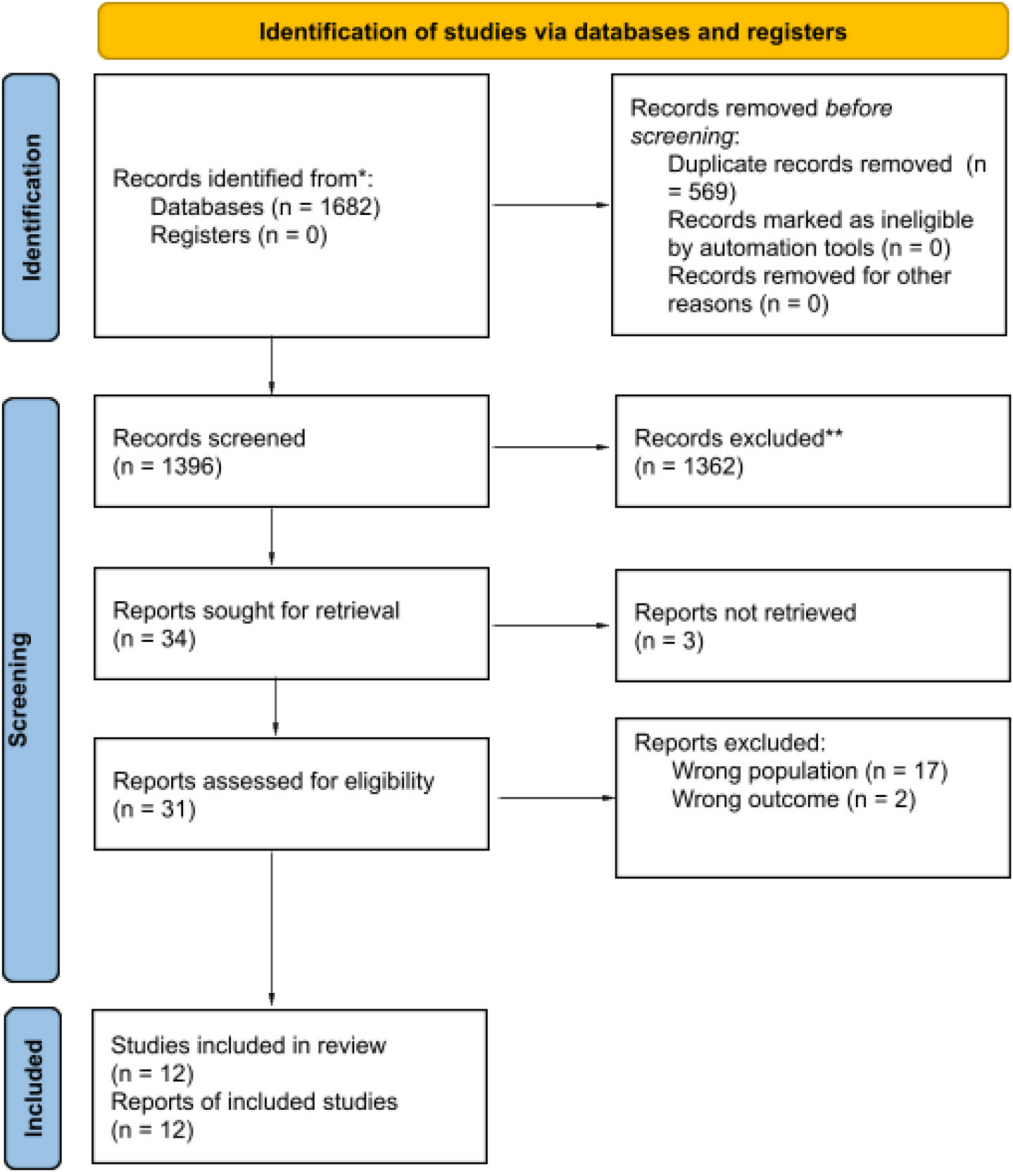
PRISMA flow diagram showing the study selection process of the systematic literature review.

A systematic review (PROSPERO registration number: CRD420251050770) was conducted to identify the studies evaluating outcomes of emergency colorectal resections in patients aged 80 years or older. PRISMA flowchart is described in Figure 1. MEDLINE (via PubMed), EMBASE, and EBSCOhost were searched for studies published from the inception of the databases to April 2025. A comprehensive literature search was performed using combinations of MeSH terms and keywords related to elderly patients, emergency colorectal surgery, mortality, postoperative outcomes, and risk or prognostic factors. The full search strategy is reported in Table 1. Study selection was performed independently by two reviewers (MB, AV), and disagreements were resolved by a third reviewer (NZ). Titles, abstracts and full-text articles were screened based on eligibility criteria in a blinded fashion using an artificial intelligence platform (Rayyan Systems Inc., Cambridge, MA). Language was restricted to English. A comprehensive PRISMA checklist was completed in accordance with established guidelines and is provided in the Supplementary Materials S5, S6. Risk of bias of the included studies were assessed using the National Health Institute (NIH) tool and reported in the Supplementary Material S4.

**Table 1:** Characteristics of studies included in the systematic review.

Author, year	Country	Study period	No. of patients >80 years, female (%)	Main indication for surgery	Cancer-related	Surgical approach	Most frequent procedure	Mortality rate	Reported predictive factors of mortality: OR/HR (95% CI)	Complication rate, reoperation rate
Leong et al. (45)	Singapore	1996–2001	58, 34 (58.6%)	Obstruction (72%)	74%	NR	Hartmann (43%)	27.5%	ASA: OR 10.4 (1.5–73.1)	81%, NR
Kurian et al. (37)	USA	2002–2009	99, NR	Perforation (33.3%)	9.1%	Open	NR	28%	Acute renal failure: OR 13 (4.0–42.6); postoperative complication: OR 8.3 (2.1–27.6); ileus: OR 3 (1.2–10.5); transfusion: OR 2 (1.3–8.6); emergent diagnosis: OR 7.2 (2.2–27.2); length of procedure: OR NR (0.00004–0.001); preoperative NH disposition: OR 3 (1.1–8.2)	NR
Kwok et al. (44)	USA	2005–2008	Validation group: 372, 237 (63.7%) Training group: 1358, 892 (65.7%)	Validation group: obstruction/perforation 96 (25.8%); training group: obstruction/perforation 282 (20.8%)	Validation group: 19.1%; Training group: 18%	93% open and 7% laparoscopic in both cohorts	NR	Validation group: 26.1%; Training group: 28.9%	Algorithm involving: Age 80–89 years: OR 0.616 (0.430,0.881); functional status (totally dependent): OR 2.539 (1.878,3.432); history of COPD: OR 1.793 (1.284,2.503); history of congestive heart failure: OR 1.872 (1.208,2.902); metastatic cancer: OR 2.000 (1.079,3.706); preoperative steroids: OR 1.610 (1.060,2.446); SIRS: OR 2.125 (1.600,2.823); creatinine 1.5 mg/dl: OR 2.572 (1.968,3.362)	Validation group: 31.3%, NR; training group: 26.9%, NR
Kolfschoten 2012	Netherlands	2010	268, NR	NR	NR	NR	NR	Stratified by no. of risk factors: 0: 7% 1: 21% 2: 41%	NR	Stratified by no. of risk factors: 0: 27%, 16% 1: 50%, 20% 2: 77%, 32%
Modini et al. (46)	Italy	2007–2009	93, 60 (64%)	Obstruction (50%)	43%	NR	Right colectomy (21.5%)	30.1%	ASA: OR 3.61 (1.69–7.31) Neurological comorbidity: OR 4.8 (1.4–16.36)	14%, NR
Ihedioha 2012	UK	2004–2009	98, 61 (62.2%)	NR	100%	NR	NR	28.6%	NR	61%, 8.2%
Zeng et al. (40)	China	2000–2019	46, 29 (63%)	NR	100%	Open (95.6%) Laparoscopic (4.4%)	Right colectomy (35.8%)	32.6%	Advanced tumor stage: HR 2.024 (1.254–3.268); ASA score ≥4: HR 2.638 (1.132–6.148); CCI >17: HR 1.784 (1.127–2.823); palliative surgery: HR 1.876 (1.011–3.481)	87%, 6.5%
El Edelbi et al. (38)	Lebanon	2010–2014	3,409, 2,105 (62%)	NR	NR	Open	NR	26%	NR	48.4%, 9.9%
Pacilli et al. (14)	Italy	2020–2022	32, 16 (50%)	Obstruction (46.8%)	37.5%	NR	NR	15.6%	NR	NR, 12.5%
Carr and NeCamp (39)	USA	2005–2021	27, NR	Obstruction (33%)	37%	NR	Sigmoidectomy (48%)	7%	NR	59%, 19%
Mathis et al. (31)	France	2015–2020	111, 68 (61.2%)	Diverticulitis (34.3%)	31.5%	Open (90.9%) Laparoscopic (9.1%)	Left colectomy (47.7%)	25.2%	Lactates: OR 1.31 (1.001–1.72) Creatinine: OR 1.0 (1.001–1.01)	51.6%, 9.9%
Kent et al. (13)	Israel	2005–2017	209, 118 (56.5%)	Obstruction (53.6%)	55%	NR	NR	37.8%	NR	NR

OR, odds ratio; HR, hazard ratio; CI, confidence interval; NR, not reported.

### Statistical analysis

Demographics and clinicopathological characteristics were summarized using counts and percentages for categorical variables, and medians with interquartile ranges (IQRs) for continuous variables. Categorical variables were compared using Chi-square or Fisher's exact test as appropriate, whereas the Mann–Whitney test was used for continuous variables. Univariate logistic regression analysis was performed to evaluate the association between individual variables and in-hospital mortality in patients 80 years or older. Variables found to be statistically significant in the univariate analysis were included in a multivariate logistic regression model to identify independent predictors of in-hospital mortality among octogenarian patients. In addition to the primary analysis focused on predictors of in-hospital mortality in octogenarians, a subanalysis was performed to compare postoperative outcomes between patients aged ≥80 and those younger than 80 years. Descriptive statistics were used to summarize the data from the systematic review. All data were analyzed using GraphPad Prism 9 (GraphPad Software Inc., CA, USA), with a *p*-value <0.05 considered statistically significant.

## Results

### Patient characteristics

During the study period, a total of 212 patients underwent emergency colorectal resections at our Institution. The cohort comprised 104 males (49%) and 108 females (51%). Patient demographic characteristics are summarized in Table 2. Group 1 included 82 patients, with a median age 85 years (IQR: 82–88, range: 80–93), while Group 2 comprised 130 patients, with a median age of 67 years (IQR: 58–74, range: 28–79).

**Table 2:** Comparative analysis of demographic, clinical and surgical variables, and postoperative outcomes between group 1 (≥80 years) and group 2 (<80 years).

Variables	Entire cohort (*n* = 212)	Group 1 (*n* = 82)	Group 2 (*n* = 130)	*p*-value
Demographics				
Age	72 (61–81)	84 (82–88)	67 (58–74)	<0.001
Gender—Female	108 (51)	48 (59)	60 (46)	0.80
Male	104 (49)	34 (41)	70 (54)	
Clinical variables				
ECOG performance status	1 (1–2)	1 (1–2)	1 (1–1)	<0.001
CCI	5 (3–6)	6 (5–7)	4 (2–5)	<0.001
ASA score	3 (2–3)	3 (3–3)	3 (2–3)	<0.001
P-POSSUM	7 (5–14)	11 (6–18)	6 (3–11)	<0.001
SIRS	72 (34)	34 (41)	38 (29)	0.06
Preoperative biochemistry				
Leukocytes (×10^9^/L)	10 (7–15)	11 (7–15)	9 (7–13)	0.35
Hemoglobin (g/L)	11.5 (10–13)	11 (9–12)	12 (10–13)	0.08
Creatinine (mmol/L)	81 (65–112)	86 (67–129)	79 (62–103)	0.07
C-reactive protein (mg/L)	23 (5–68)	25 (5–55)	23 (6–66)	0.90
Albumin (g/dl)	30 (24–33)	26 (21–30)	30 (24–33)	0.04
Lactate (mmol/L)	1.5 (1–3)	1.5 (1–2)	1.6 (1–4)	0.50
Indication for surgery				
Perforation	94 (44)	26 (32)	68 (52)	0.006
Obstruction	80 (38)	35 (43)	45 (35)	0.12
Bleeding	14 (7)	10 (12)	4 (3)	0.001
Ischemia	24 (11)	11 (13)	13 (10)	0.50
Etiology				
Malignant	94 (44)	46 (56)	48 (37)	0.006
Surgery classification				
Emergent (<12 h)	55 (26)	22 (27)	33 (25)	0.80
Urgent (12–17 h)	76 (36)	27 (33)	49 (38)	
Delayed (>72 h)	81 (38)	33 (40)	48 (37)	
Postoperative outcomes				
Clavien-Dindo	1 (1–2)	1.5 (1–2)	1 (0–2)	0.16
Clavien-Dindo ≥III	52 (25)	18 (22)	34 (26)	0.48
Planned reoperation	13 (6)	5 (6)	8 (6)	0.003
Reoperation for complications	11 (5)	1 (1)	10 (8)	0.05
LOS, days	12 (8–18)	14.5 (10–22)	12 (8–17)	0.003
30-days mortality	20 (9)	11 (13)	9 (7)	0.33
In-hospital mortality	30 (14)	20 (24)	10 (8)	<0.001

Data are expressed as No. (%) or median (IQR) or mean (range).

ECOG, Eastern Cooperative Oncology Group; CCI, Charlson Comorbidity Index; ASA, American Society of Anesthesiologists; P-POSSUM, Portsmouth Physiological and Operative Severity Score for the enUmeration of Mortality and Morbidity; SIRS, Systemic Inflammatory Response Syndrome; LOS, Length Of Stay.

Group 1 exhibited a preoperative EGOG score significantly higher than Group 2 [1 (IQR: 1–2) vs. 1 (IQR: 1–1); *p* < 0.001]. The age-adjusted CCI was more elevated in octogenarians (median 6, IQR: 5–7) compared to younger patients (median 4, IQR: 2–5) (*p* < 0.001). Predicted mortality risk as estimated by P-POSSUM was significantly greater in Group 1 (median 11, IQR: 6–18) than in Group 2 (median 6, IQR: 3–11) (*p* < 0.001). The frequency of SIRS did not differ appreciably (41% vs. 29%, respectively).

Among patients aged ≥80 years, the most common admission diagnosis was bowel obstruction (43%), followed by perforation (32%), colonic ischemia (13%), and bleeding (12%). In the younger cohort, colonic perforation (52%) was significantly predominant (*p* < 0.001), then obstruction (35%), ischemia (10%) and bleeding (3%) also occurred. Acute diverticulitis was diagnosed in 28 patients (34%) in Group 1 and in 52 patients (40%) in the younger group. A malignant tumor was identified in 56% of octogenarians and 37% younger patients. Characteristics of cancers in our cohort are summarized in Supplementary Material S1. Across the entire cohort, 55 patients (26%) underwent surgery within 12 h of admission, 76 (36%) between 12 and 72 h, and 81 (38%) after more than 72 h. The open surgical approach was the most frequently used, being performed in 63 patients (77%) in Group 1 and 77 patients (59%) in Group 2, whereas the laparoscopic approach was adopted in 19 octogenarians (23%) and 53 younger patients (41%). In both groups, the most frequently performed procedure was Hartmann's procedure (37% in Group 1, 44% in Group 2). An ostomy was created in 40 octogenarians (49%) and in 80 patients from the younger cohort (64%). The surgical procedures performed are summarized in the Supplementary Material S2. Postoperative complications classified as Clavien-Dindo grade ≥III were observed in 18 patients (22%) in Group 1 and 34 patients (26%) in Group 2, without a statistically significant difference between the groups (*p* = 0.48). Postoperative complications are reported in Supplementary Material S3. Group 1 showed longer length of stay (LOS) and higher 30-day mortality, considering both in-hospital and post-discharge deaths. Specifically, the median LOS was 14.5 days (IQR: 10–22) in the elderly cohort vs. 12 days (IQR: 8–17) in the younger group. In our series, reoperation for staged laparotomy occurred in 5 patients (6%) in Group 1 and in 8 patients (6%) in Group 2, whereas reoperation for surgical complications occurred in 1 patient (1%) in Group 1 and in 10 patients (8%) in Group 2.

Differently, in-hospital mortality rate was significantly higher in the octogenarian group compared to the younger cohort (*p* < 0.001), occurring in 20 patients (24%) and 10 patients (8%), respectively. Patients' characteristics are summarized in Table 2. The median follow-up was 10 months (range: 1–75 months).

### Univariate and multivariate predictive analysis of in-hospital mortality in octogenarians

In univariate analysis, higher ASA score (OR: 2.89, 95% CI: 1.27–6.49, *p* = 0.01), use of antithrombotic drugs (OR: 4.42, 95% CI: 1.03–13.12, *p* = 0.01), elevated serum lactate levels (OR: 1.33, 95% CI: 1.04–1.69, *p* = 0.02), intraoperative administration of vasoactive agents (OR: 0.33, 95% CI: 0.12–0.79, *p* = 0.01) and blood transfusions (OR: 3.53, 95% CI: 1.39–8.41, *p* = 0.005) were associated with in-hospital mortality. Multivariate logistic regression underlined that only high lactate serum level was an independent negative prognostic factor for in-hospital mortality in octogenarian patients (OR: 2.38, 95% CI: 1.26–5.31, *p* = 0.01). Univariate analysis is reported in Table 3 and multivariate analysis in Table 4.

**Table 3:** Univariate analysis of variables associated with in-hospital mortality in group 1 (≥80 years).

Variables	In-hospital mortality: Yes (*n* = 20)	In-hospital mortality: No (*n* = 62)	OR (95% CI)	*p*-value
Preoperative variables				
Age	83 (82–87)	84 (82–88)	0.91 (0.78–1.05)	0.19
Gender—Female	9 (45)	25 (40.3)	1.21 (0.44–3.11)	0.79
Male	11 (55)	37 (59.7)		
ASA score				
I	0	0	2.89 (1.27–6.49)	**0**.**01**
II	2 (10)	7 (11.3)		
III	12 (60)	49 (79)		
IV	6 (30)	6 (9.7)		
ECOG performance status	2 (1–2.5)	2 (1–2)	1.09 (0.73–1.64)	0.65
CCI	6 (5–7)	6 (4–7)	0.85 (0.71–1.11)	0.39
P-POSSUM	35 (31–38)	37 (34–41)	1.28 (0.97–1.11)	0.19
SIRS	8 (40)	18 (29)	1.60 (0.63–3.82)	0.29
Antithrombotic drugs	3 (15)	2 (3.2)	4.42 (1.03–13.16)	**0**.**01**
Comorbidities				
Cardiovascular	18 (90)	47 (75.8)	1.01 (0.99–1.19)	0.6
Pulmonary	1 (5)	9 (14.5)	0.31 (0.02–1.51)	0.25
Hepatic	2 (10)	5 (8.1)	1.31 (0.81–3.67)	0.78
Renal	2 (10)	13 (21)	0.5 (0.08–1.72)	0.35
Oncological	5 (25)	20 (32.3)	0.67 (0.22–1.72)	0.44
Cerebrovascular	18 (90)	47 (75.8)	1.05 (0.34–2.69)	0.91
Hematological	0 (0)	3 (4.8)	0 (0–1.71)	0.15
Metabolic	1 (5)	15 (24.2)	0.22 (0.01–1.03)	0.14
Preoperative biochemistry				
Leukocytes (×10^9^/L)	13 (9–15)	8 (6–13)	1.01 (0.98–1.11)	0.14
Hemoglobin (g/L)	11 (10–13)	11 (9–13)	1.05 (0.86–1.23)	0.67
Creatinine (mmol/L)	114 (67–176)	85 (66–176)	1.51 (1.01–1.21)	**0**.**05**
C-reactive protein (mg/L)	24 (7.3–140)	36 (22–100)	0.99 (0.98–1.23)	0.91
Albumin (g/dl)	25 (21–29)	28 (22–31)	0.94 (0.79–1.07)	0.34
Lactate (mmol/L)	2.6 (1–7)	1.5 (1–2.7)	1.33 (1.04–1.69)	**0**.**02**
Indication for surgery				
Perforation	6 (30)	20 (32.3)	1.3 (0.23–1.33)	0.16
Obstruction	6 (30)	29 (46.7)	0.55 (0.22–1.32)	0.18
Bleeding	3 (15)	7 (11.3)	0.79 (0.08–1.91)	0.42
Ischemia	5 (25)	6 (9.7)	1.2 (0.28–1.49)	0.10
Etiology				
Malignant	9 (45)	35 (56.5)	1.62 (0.19–1.15)	0.11
Benign	11 (55)	27 (43.5)		
Operative and postoperative variables				
Operative time, min	172 (141–212)	205 (142–244)	1.01 (0.98–1.02)	0.31
IO vasoactive agents	14 (70)	22 (35.5)	0.33 (0.12–0.79)	**0**.**017**
IO blood transfusion	8 (40)	7 (11.3)	3.53 (1.39–8.41)	**0**.**005**

Data are expressed as No. (%) or median (IQR).

Bold values indicate statistical significance (*p*-value < 0.05).

ASA, American Society of Anesthesiologists; ECOG, Eastern Cooperative Oncology Group; CCI, Charlson Comorbidity Index; P-POSSUM, Portsmouth Physiological and Operative Severity Score for the enUmeration of Mortality and Morbidity, SIRS: Systemic Inflammatory Response Syndrome; IO, Intraoperative.

**Table 4:** Multivariate logistic regression analysis of independent predictors of in-hospital mortality in Group 1 (≥80 years).

Variables	Odds ratio	95% CI	*p*-value
ASA score	1.81	1.7–12.9	0.07
Anticoagulants	1.4	0.06–3.16	0.16
Creatinine	1.36	0.97–1.01	0.17
Lactate	2.38	1.26–5.31	**0**.**01**
IO vasoactive agents	0.4	0.17–18.9	0.69
IO blood transfusion	1.60	0.65–10.39	0.1

Bold values indicate statistical significance (*p*-value < 0.05).

CI, confidence interval; ASA, American Society of Anesthesiologists; IO, intraoperative.

### Systematic review results

A total of thirteen retrospective cohort studies published between 2009 and 2023 were included in the review and are summarized in Table 1. Supplementary Figure S1 illustrates the PRISMA flow diagram showing the study selection process. Risk of bias assessment is shown in Supplementary Material S4. Studies conducted in the USA, France, Italy, Singapore, Israel, the UK, China, the Netherlands, and Lebanon were included. All studies involved octogenarian patients undergoing emergency colorectal resections, and all of them were retrospective cohort studies. Sample sizes ranged from 27 to 3,409 patients, with the proportion of females varying between 50% and 65%. The most common indications for surgery were colon obstruction and acute diverticulitis, with cancer-related conditions reported from 31% to 100% of cases. Thirty-day mortality ranged from 7% to 41%, while complication rates from 14% to 87%. Reoperation rates varied between 9% and 32%. Several studies identified significant predictors of mortality, including elevated lactate levels, impaired renal function, advanced tumour stage, CCI, palliative surgery, neurological comorbidities, and higher ASA scores.

## Discussion

Colorectal resections account for 7% of all emergency surgeries performed annually at Our Department, and in literature it is reported that 23.5% of colorectal cancer surgeries are performed in emergency settings (22). A high percentage of these interventions involved octogenarian patients (39%), a group with well-documented susceptibility to postoperative complications, which further contribute to the increased morbidity and mortality inherently associated with emergency surgical procedures (23). This disparity is primarily attributable to life-threatening nature of the underlying conditions, the frequent occurrence of physiologic derangements at presentation, and the need to operate under time pressure, frequently during night shifts or in suboptimal clinical settings (8). The main goal of surgeons in this scenario is to offer the most appropriate treatment with clinical benefit and the improvement of the residual quality of life. In selected patients, less invasive procedures such as stoma formation or endoscopic stents placement may be considered as safer alternatives to definitive resection, avoiding the risks associated with extensive surgical procedures (24). A comprehensive preoperative risk assessment is essential in case of high risk patients, thus, it is interesting to know how outcome prediction in this patient population could be improved and, more importantly, how their perioperative management could be optimized. The aim of this study was to identify short-term mortality predictors in elderly patients undergoing colorectal resections in emergency scenarios, enhancing surgical decision-making and optimizing patient management. Moreover, to our knowledge, this is the first systematic literature review specifically addressing outcomes and predictive factors in patients aged 80 years or over undergoing emergency colorectal resections.

Among octogenarians, our data showed an in-hospital mortality rate of 24%, which is significantly higher than in the younger group (8%). Our findings are consistent with those from previously published studies. A total of 12 studies were included in our systematic literature review, reporting in-hospital mortality rates among octogenarians ranging from 7% to 41% (13, 14). Higher mortality rate among octogenarians may be attributed in part to the frequent presence of atypical symptoms, such as generalized weakness, confusion, or mild abdominal discomfort, rather than the classic signs of acute surgical conditions, which can complicate clinical assessment and mask serious conditions, leading to delayed diagnosis (25).

Additionally, as confirmed in our analysis, octogenarians typically exhibited higher values of preoperative classical frailty markers such as ASA, ECOG, CCI, and P-POSSUM (26). However these variables do not reach statistical significance as prognostic factors in our study. This may be explained by an accurate preoperative selection with the promotion of non operative management and palliative care for the more severely ill patients.

By analyzing the cause of admission in our cohort we observed that octogenarians show higher incidence of obstruction (43%) and malignancy (56%). These findings align with existing literature, where colonic obstruction was the most frequently reported surgical indication in 5 of the 12 studies included in our systematic review (range: 33%–72%) and is commonly associated with cancer, which was identified in a substantial proportion of patients among those studies that explicitly reported malignancy rates (range: 9%–100%).

The high prevalence of emergency presentations of malignancy (56%) in our octogenarian group further highlights the importance of early detection and timely diagnosis of colorectal cancer. In this context, recent studies have investigated the integration of deep learning algorithms into clinical practice for the classification and diagnosis of CRC histopathology images, showing promising potential to enhance both the accuracy and efficiency of CRC detection (27, 28).

In contrast, the younger cohort shows a predominance of colonic perforations (52%) primarily related to acute diverticulitis (40%). This trend reflects the results reported by other authors (29, 30) in which perforated acute diverticulitis was significantly higher in young patients. Among octogenarians in our cohort, acute diverticulitis was identified in 28 patients (34%), a finding that is consistent with the results of our systematic review (31). In the elderly, as confirmed in our analysis, lower gastrointestinal bleeding was observed more frequently. Aging, as known, is associated with several changes in the gastrointestinal tract, including decreased mucosal perfusion, diminished regenerative capacity, and increased susceptibility to ischemia (32). Moreover, typically elderly present with higher rates of renal impairment and more frequent use of antithrombotic drugs (OR: 4.42, 95% CI: 1.03–13.16; *p* = 0.01). These factors make these patients particularly susceptible to bleeding complications, especially in the perioperative setting (33).

Wound infections—among the most common complications following colorectal surgery and a significant source of pain and morbidity for patients with a negative impact in terms of morbidity, LOS, economic impact, readmission and sepsis (34)—have been reported in the literature with an incidence of approximately 12% (35). In our cohort, surgical site infections (SSIs) were observed in 4 patients (5%) in the octogenarian group and in 8 patients (6%) overall, as detailed in Supplementary Material S3. This relatively low rate of SSIs is likely influenced by the limited sample size of our study and may also be partially explained by the frequent use of negative pressure wound therapy in this patient population. Additionally, although this parameter was not assessed in our study, recent research in this field has identified a correlation between reduced levels of butyrylcholinesterase (BChE)—a non-specific cholinesterase enzyme predominantly found in the liver and other tissues, and typically associated with systemic inflammation—on postoperative days 1 and 3, and an increased risk of surgical site infections (SSIs) (36).

Interestingly, in our study 19 octogenarians (23%) underwent laparoscopic surgery, a markedly higher rate compared to what is reported in the literature, where a higher percentage of emergency procedures in this population are typically performed via an open approach (37, 38). This finding underscores that, when appropriately applied in carefully selected patients, laparoscopic surgery does not compromise surgical outcomes in emergency settings. Nonetheless, in these patients an open approach is often preferred because of the increased anesthetic risk associated with their deteriorated clinical condition.

Reoperation is sometimes required to manage surgical complications or in the context of an open abdomen, where a second-look procedure is already planned.

In our series, major complications occurred in 18 patients (22%) among octogenarians, and only one of them required reoperation for evisceration, which is notably lower than the rates reported in previous studies (range: 6%–19%) (39, 40). These findings may be partially explained by the high number of stomas performed, a factor known to reduce the risk of postoperative complications, including anastomotic leakage. Additionally, management by a dedicated acute care surgery team, as in Our Institution, where surgeons are formally certified in acute care surgery, has been independently associated with a lower overall rate of postoperative complications compared to care provided by general surgeons alone (41). However, this population remains at increased risk of morbidity, therefore, treatment strategies should be individually tailored based on a comprehensive evaluation of the patient's clinical condition.

In our multivariate analysis, hyperlactatemia (>2 mmol/L) resulted as an independent predictor of short-term mortality in octogenarians. Mathijis et al. (31) first described this correlation: in their analysis hyperlactatemia (OR: 0.03; 95% CI: 1.00–1.72) and hypercreatininemia (OR: 1.00; 95% CI: 1.00–1.01) emerged as independent predictors of early postoperative mortality following emergency colorectal surgery in octogenarians. Notably, high lactate levels reflect tissue hypoperfusion and increased anaerobic metabolism, accounting for its association with severe clinical conditions such as hemorrhage, sepsis, ischemia or multi-organ failure (42, 43). Nevertheless, lactate levels are not routinely evaluated in every emergency department. Therefore, it is fundamental to emphasize their important role in the emergency setting, particularly in frail patients. Our analysis showed that hypercreatininemia was significantly associated with mortality in the univariate analysis, but did not remain significant when adjusted for other variables. This result could be explained by the small sample of impaired renal function in our cohorts among non-survivors and survivors (10% vs. 21%). Nonetheless, the association at univariate analysis confirms the pivotal role of this biomarker as a predictor (31, 44).

Preoperative comorbidity assessment, as measured by the ASA score, has also been previously described as a short-term prognostic factor in this population. In a study by Leong et al., 55% of patients aged ≥80 years undergoing emergency colorectal surgery had an ASA score ≥3, which significantly increased their risk of short-term mortality (45). Similarly, Modini et al. identified a high ASA grade as a significant risk factor in older patients (46), while another study further associated advanced tumor stage, palliative surgery, ASA score ≥4, and a CCI >17 with poorer outcomes (41). In our study, many patients, regardless of age group, were classified as ASA score ≥3 and this value was highly prevalent (90%) among non-survivors octogenarians, reaching significance only at univariate. In emergency scenarios most patients typically present with acute pathological conditions that, irrespective of baseline health status, justify classification as having a severe systemic disease with functional limitations. As such, the ASA score in this setting may be more indicative of the acute surgical indication rather than underlying chronic comorbidities, thereby limiting its discriminatory value across age groups.

The main limitation of this study is its retrospective, single-centre design, although data were retrieved from a prospectively maintained database. A larger patient cohort and longer follow-up period would also be beneficial to assess short- and long-term outcomes. This review is limited by the inclusion of only English-language studies. Moreover, all included studies were retrospective in nature, which may introduce inherent biases.

Nevertheless, this study provides important insights to improve the management of a population increasingly encountered in contemporary hospital settings. Reviewing comparable studies enables assessment of clinical outcomes and highlights the tools available to the emergency surgeon to optimize them.

Elderly often compels surgeons to make life-saving decisions in complex situations, where a clear clinical assessment is not always feasible. Nevertheless clinical evaluation remains fundamental in determining the appropriate therapeutic strategy, but a comprehensive patient assessment can be particularly helpful in borderline situations. As previously reported, ASA score, hypercreatininemia, and elevated lactate levels are among the most consistently described predictors of mortality in the literature. These factors must be carefully evaluated when treating elderly patients who present with limited disease and maintain an otherwise acceptable clinical status. In certain scenarios these predicting factors suggest a markedly increased short-term mortality risk, supporting less invasive procedures when feasible.

## Conclusions

This study highlights the elevated risk of short-term mortality among octogenarian patients undergoing emergency colorectal resections. Adequate preoperative assessment is essential to ensure the safety of these procedures, and early identification of prognostic factors, such as serum lactate levels, can enhance patient selection and optimize perioperative management.

The presence of a specific acute care surgery department has led to an awareness of these situations, finally improving clinical outcomes.

## Data Availability

The raw data supporting the conclusions of this article will be made available by the authors, without undue reservation.
